# Improving predictive models for Alzheimer’s disease using GWAS data by incorporating misclassified samples modeling

**DOI:** 10.1371/journal.pone.0232103

**Published:** 2020-04-23

**Authors:** Brissa-Lizbeth Romero-Rosales, Jose-Gerardo Tamez-Pena, Humberto Nicolini, Maria-Guadalupe Moreno-Treviño, Victor Trevino

**Affiliations:** 1 Tecnologico de Monterrey, Escuela de Medicina y Ciencias de la Salud, Monterrey, Nuevo Leon, Mexico; 2 Genomics of Psychiatric and Neurodegenerative Diseases Laboratory, National Institute of Genomic Medicine (INMEGEN), Mexico City, Mexico; 3 Departamento de Ciencias Basicas, Universidad de Monterrey, San Pedro Garza Garcia, Nuevo Leon, Mexico; Korea National University of Transportation, REPUBLIC OF KOREA

## Abstract

Late-onset Alzheimer’s Disease (LOAD) is the most common form of dementia in the elderly. Genome-wide association studies (GWAS) for LOAD have open new avenues to identify genetic causes and to provide diagnostic tools for early detection. Although several predictive models have been proposed using the few detected GWAS markers, there is still a need for improvement and identification of potential markers. Commonly, polygenic risk scores are being used for prediction. Nevertheless, other methods to generate predictive models have been suggested. In this research, we compared three machine learning methods that have been proved to construct powerful predictive models (genetic algorithms, LASSO, and step-wise) and propose the inclusion of markers from misclassified samples to improve overall prediction accuracy. Our results show that the addition of markers from an initial model plus the markers of the model fitted to misclassified samples improves the area under the receiving operative curve by around 5%, reaching ~0.84, which is highly competitive using only genetic information. The computational strategy used here can help to devise better methods to improve classification models for AD. Our results could have a positive impact on the early diagnosis of Alzheimer’s disease.

## Introduction

Alzheimer’s Disease (AD) is the most common form of dementia in the elderly, accounting for 60–70% of these cases [1]. AD affects memory, thinking, behavior, and in general, the ability to perform daily activities. According to the World Health Organization, around 50 million people have dementia, and there are about 10 million new cases every year. The disease has two subtypes based on the age of onset: early-onset AD (EOAD) and late-onset AD (LOAD). EOAD is present in around 5% of cases [2], with an age-onset from the 30s to mid-60s. The genes associated are presenilin 1 (PSEN1), presenilin 2 (PSEN2), and amyloid precursor protein (APP) [3]. On the other hand, LOAD occurs after the mid-60s and has 90–95% of incidence [2]. The most common gene confirmed as a risk factor is apolipoprotein E (APOE e4) [4]. LOAD seems to be a more complex disorder that is caused by both genetic and environmental factors [5]. Since AD has no cure [4,6], understanding the genes involved in the evolution of the disease will serve as a guide for early diagnosis to identify subjects at risk for closer follow up, treatment, and prevention. In this context, computational methods to improve diagnosis or identify novel gene candidates are highly appreciated.

Genome-Wide Association Studies (GWAS) have played an important role in AD by measuring and analyzing single nucleotide polymorphism (SNP) markers across the human genome [7]. The analysis consists of detecting differences in the frequency of variations among populations. Traditional methods for analyzing GWAS data, such as the χ^2^ test and the logistic regression, are based on statistical techniques that evaluate variables one by one for their ability to discriminate between groups of samples (i.e., cases vs. controls) [8]. These univariate methods have been successful in identifying hundreds of genomic regions. Nevertheless, a substantial fraction of the phenotype heritability remains unexplained [9]. Possible explanations include that interactions between markers when predicting a disease are not considered [10], and the use of strict p-value controls over the candidate SNPs [11].

Several studies have analyzed GWAS data for AD trying to identify novel genes and variants. For example, in 2013, a meta-analysis of datasets from 4 large consortium and 11 countries, including ~17,000 AD cases, identified around 20 loci [12]. These loci increased to 25 and 29 in 2019, implicating around 200 genes in two meta-analyses of ~70,000 AD cases [13],[14]. Here, we used a subset of AD samples as a proof of concept.

As a complex disease, AD could be influenced by multiple genetic variants. In general, neurodegenerative disorders are a challenge in terms of prediction because pathological information cannot be easily accessed [15]. To face this issue, practical and non-invasive solutions must be developed to help in the diagnosis. Thus, multivariate approaches are interesting to be explored because they intrinsically consider interactions and combinations between SNPs and aggregate putative small effects to achieve higher predictive power [16].

There are many approaches to generate multivariate predictive models from GWAS data [17]. The polygenic risk score (PRS) is the most popular approach and has been applied to AD several times [17–22]. PRS is a sum of univariate estimated coefficients; thus, it is easily interpreted. For AD, the predictive classification power, in terms of the area under the receiving operative curve (AUC), has been estimated from 0.68 to 0.83 [18–20]. The results depend on data included, such as APOE status, mild cognitive impairment (a hypothetical pre-stage for AD), the AD type (LOAD or EOAD), and, more importantly, the clinical information used such as age, gender, and other data. The most recent meta-analysis used a PRS whose AUC was 0.827 but included SNPs barely associated, from p = 0 to p < 0.5 [14]. In principle, their PRS included thousands of SNPs and therefore, could be overestimated (the precise number of SNPs is not mentioned).

Rather than PRS, other approaches can be used that have the potential to provide better results [17]. The application of Machine Learning (ML) techniques based on genetic data has been popularized to identify risk factors associated with several diseases since they have proven to be robust when the problem involves hundreds of thousands of predictors [23–25]. The main idea in ML is building predictive models maximizing the accuracy of classification between patients and controls. However, the problem of having hundreds of thousands of markers from GWAS data and only a few thousands of samples remain a computational limitation. Feature selection methods face the problem of having many variables by reducing the number of irrelevant or redundant variables and keeping the ones more relevantly associated with outcome [23,25]. By applying feature selection techniques, further computation becomes lighter, providing a better understanding of the problem and, generally, increasing the prediction accuracy [23,25]. Pre-selection can be done by univariate approaches testing various cut-off criteria resulting in varied subset sizes, and then subsets are coupled to ML techniques improving the area under the curve (AUC). This has been tested in type 1 diabetes [26], Crohn’s Disease [27], and Genitourinary Toxicity [16]. Other methods involve more robust algorithms such as LASSO and stepwise, coupled to ML methods. For example, LASSO was applied to duloxetine response in major depressive disorder [28], and stepwise logistic regression applied to aid the diagnosis of dementia [29]. In particular, LASSO uses a linear model corrected by a regularization parameter providing feature selection and model building simultaneously [30] while in stepwise, the best variable is chosen and added to the model at each step. Also, other more sophisticated methods, such as genetic algorithms, have also been applied to GWAS data of bipolar disorder [31].

To improve upon current approaches applied to GWAS in AD, we assumed that original samples are hiddenly stratified by SNP of a more subtle association within a small fraction of patients. Thus, in this work, we improved the classical analysis pipeline by adding a statistical modeling step of misclassified samples and compared three multivariate machine learning strategies including LASSO, a stepwise algorithm (BSWiMS) [32] and genetic algorithms [33] for their ability to build multivariate models in classical pipelines using GWAS data only. To decrease the variable space, we used SNP selection based on the χ^2^ test after rigorous quality control processing applied to GWAS data from Alzheimer’s disease, specifically from LOAD. We show that LASSO provides better results than the other methods and that the additional misclassification modeling improves substantially the classification accuracy and AUC, which might also provide interesting SNP that would otherwise be missing. To the best of our knowledge, this is the first analysis comparing LASSO and other multivariate approaches in GWAS data from AD and also the first approach incorporating misclassification models. Our strategy and results may have an impact on the early diagnosis of AD.

## Materials and methods

**Fig 1:**
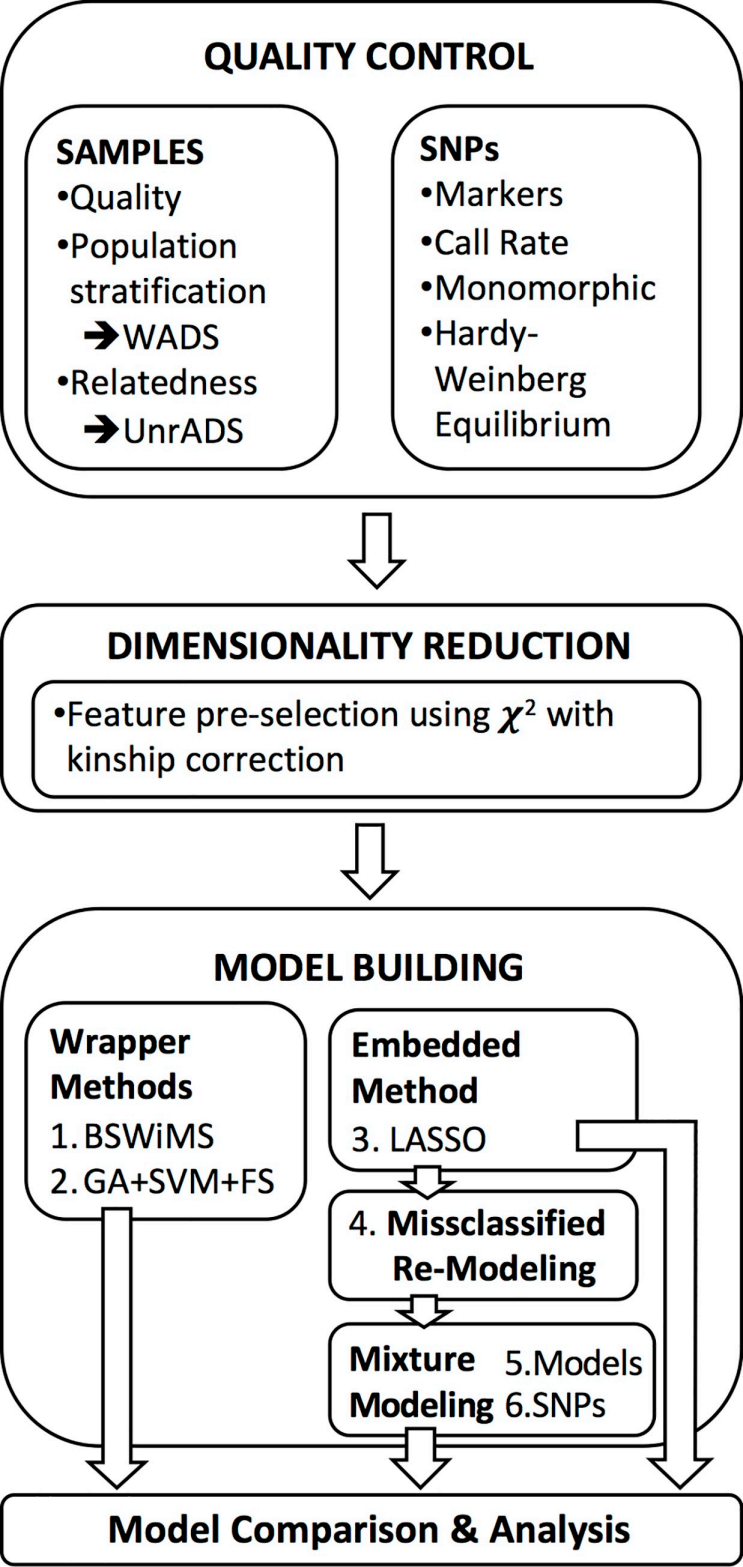
Schematic representation of the analysis.

The main objective of this research is to improve classification accuracy and extend the set of possible genetic risk factors for AD. Overall, the methodology of this research (Fig 1) includes procedures to obtain clinical and genotypic data, to filter data based on quality control criteria, to reduce dimensionality, to build statistical models, and to analyze and compare the models.

### Clinical and polymorphism data

The data used in this research correspond to the National Institute on Aging—Late-Onset Alzheimer’s Disease Family Study: Genome-Wide Association Study for Susceptibility Loci (phs000168.v2.p2) and were formally requested and approved from the National Center for Biotechnology Information (NCBI) through the Genotypes and Phenotypes database (dbGaP) [34]. The study has four groups accounting for 5,220 individuals and 620,901 SNPs. The cases were diagnosed with *Definite* AD by neuropathological criteria or *Probable* AD by NINCDS-ADRDA criteria, and the age at diagnosis should be equal to or greater than 60 years. The controls were defined as individuals without manifestations of cognitive deterioration or memory loss through neuropsychological tests in addition to not having a history of psychiatric or neurological disorders. More details can be found in the seminal study [34].

### Quality control for subjects

Recommended GWAS quality control (QC) was tightly followed [35]. In addition, it is known that populations show differences in allele frequencies due to ancestry diversity, which can cause spurious results in association studies [35]. As shown in S1A Fig, from the initial 5,220 subjects, we removed those having an unclear phenotype (n = 659). Then, we removed those non-European-Americans subjects (n = 705). For this, we used a principal component analysis (PCA) implemented with the EIGENSTRAT method [36,37] using 100,000 randomly chosen SNPs controlling for chromosome size. The populations were selected from the clusters formed by the first two PC basically by selecting a threshold in the first PC that discriminates between clusters, commonly close to zero. The data was processed with the AssocTests package [38] of the statistical software R [39]. The filtering by population stratification was performed separately in cases and controls.

### Datasets generation

The QC procedure generated 1,856 AD cases and 2,000 controls (S1A Fig). Because this dataset contains all subjects, it will be referred to as *Whole Alzheimer’s Disease Set* (WADS). This dataset was used to pre-select SNPs before the multivariate analysis. Nevertheless, the data used in this study correspond to a family study in which there were up to two relatives per affected individual. If subjects are treated as independent samples, the results can bias the study increasing false positives and false negatives [35]. For this reason, only one member per family was randomly selected, with the information provided in the pedigree data (pht000709.v2.p2) [34]. A repetition was performed to analyze the effect on the model. The resulted dataset will be referred to as *Unrelated Alzheimer’s Disease Set* (UnrADS). This dataset was used to perform predictive models, and consist of 1,830 individuals, of which 813 were AD cases and 1,017 were controls.

### Quality control for SNP data

The overall SNP quality process is shown in S1B Fig. Briefly, the original SNP dataset generated by the Center for Inherited Disease Research (CIDR) included 620,901 markers using the Illumina Infinium II assay protocol with hybridization to Illumina Human 610Quadv1 B Beadchips was processed to select only autosomal chromosomes, so the markers of chromosome X and Y were removed. The remaining were 600,470 SNPs. Then, we removed ~3% markers that did not have a reference SNP ID number (rs), leaving a set of 582,539 SNPs. We also removed markers having a call rate lower than 98% [37], resulting in 2.5% SNPs from controls or 2.4% from patients. Monomorphic markers were also removed (having the same genotype in all subjects). SNPs deviated from Hardy-Weinberg Equilibrium (HWE) with a p-value less than 10^−5^ (0.17% of the markers) were removed as suggested [37]. The use of these filters of cases and controls resulted in 561,309 high-quality SNPs.

### Dimensionality reduction by feature pre-selection

For the multivariate model building, we filtered features using a univariate test to select those more promising SNP markers. Since the data used in this research comes from a family study, an approach suggested by [40] was considered to correct for relatedness by estimating kinship coefficients in a χ^2^ test. For this, we used the dataset WADS applying the χ^2^ test to each SNP and removing those whose *p*-value was less than a threshold. Two thresholds were used.

### Machine learning models

This study proposes the use of machine learning methods to build predictive models that maximize discrimination between healthy individuals and individuals with AD based solely on GWAS data. The advantages of ML methods against traditional techniques are their ability to consider interactions between features and exploring not obvious combinations. For the model building, wrapper and embedded methods were tested.

The first wrapper method used was Bootstrapping Stage-Wise Model Selection (BSWiMS) [32], which is based on statistics and procedures of forward and backward selection to generate a logistic model. This method uses 20 cycles of internal cross-validation. The second wrapper method used, GALGO, is based on stochastic searches using an ensemble of Genetic Algorithms (GA) coupled to a Support Vector Machine (SVM) classifier followed by a Forward Selection (FS) [33]. Commonly, hundreds or thousands of models are built, then a final representative model is generated by FS depending on feature frequency. The third algorithm used is LASSO [30], one of the most well-known embedded methods, which use L1-regularization that performs a feature selection process in the training stage of the model. LASSO was ran using the FRESA.CAD package. The error estimation was estimated performing 20 rounds of internal cross-validation (CV) to 80% of the dataset for training and 20% for testing. For this, the lambda parameter was first tuned using the average of a 10-fold CV to obtain the minimum mean cross-validated error. The classification accuracies and area under the receiving operative curve (AUC) were estimated from the performance on the blind test set. Because the above process generates 20 models, a representative LASSO model was estimated re-running LASSO on the first training set. This is not an issue because two runs on different sets generate very similar results, as shown in the results section.

To improve models, we added a second modeling step for those samples wrongly classified in the first step. This assumes that wrongly classified samples pertain to non-learned sub-classes, which are characterized by specific SNPs. The feature pre-selection and model building were re-run. Then, we compared two methods for building the final model. The first method involves adding the selected SNPs directly to the original model, while the second method involves adding the pre-selected SNPs to the pool of SNPs before model building. This procedure was performed only for LASSO because it was the method with the best performance in the first step.

For all model building experiments, we used the dataset UnrADS. Although the missing values were low (0.078% in Top100 and 0.093% in T1k), imputation was performed by assigning the median value of the nearest neighbors. This was performed using the function *nearest Neighbor Impute()* of FRESA.CAD that contains the BSWiMS [32].

## Results

### Data validation

**Fig 2:**
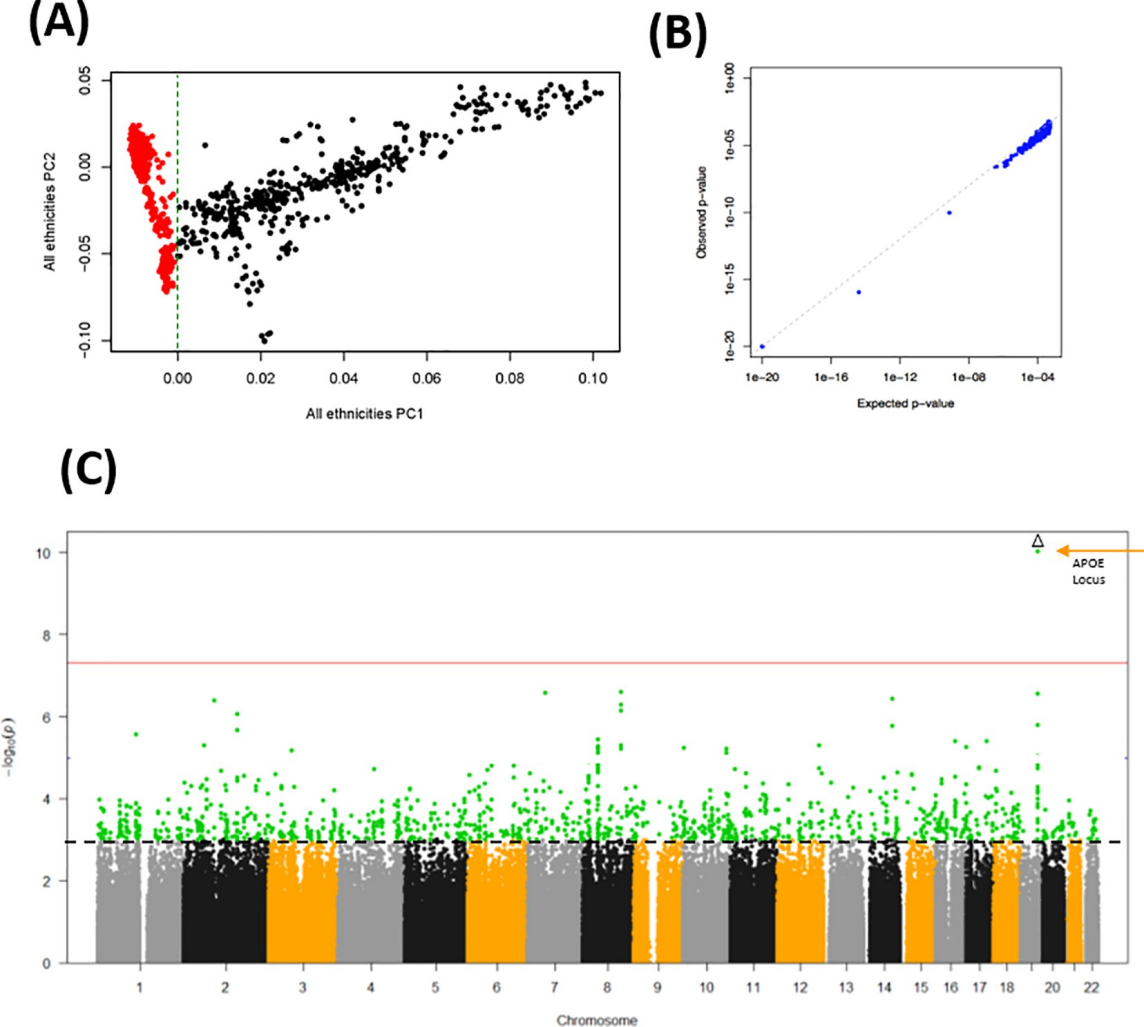
Validation of the data processing procedure. (A) First two principal components representing ethnic clusters. Red dots represent patients of European origin. (B) Validation of p-values compared to those from a reference study. (C) Manhattan plot of the obtained p-values in -Log10 scale. APOE locus is indicated. The red line shows genome-wide significance. The dotted black line marks a reference at 10^−3^. Because of the large -Log10 p-values of the APOE locus, the overall scale has been reduced for clarity.

To confirm that our data processing was valid, we compared our subject selection with that processed elsewhere [37]. Our principal components result effectively confirm that subject selection for European-Americans was correct (Fig 2A). Likewise, the p-values estimated from our analysis using the corrected χ^2^ test also concur with those p-values available in a previous study (Fig 2B). The obtained p-values are shown in Fig 2C. As expected, SNPs close to APOE on chromosome 19 show the stronger genome-wide significant association with AD. Overall, these results show that our data processing display expected and comparable results and are therefore valid for further analyses.

### SNP Pre-selection

The conventional genome-wide significant p-value threshold of 5×10^−8^ seems too conservative, selecting very few SNPs. Moreover, recent comparisons suggest less restrictive significance thresholds to achieve higher power [11]. In addition, we would like to explore multivariate scenarios where combinations of SNP, rather than SNP on its own, are possibly associated with disease by achieving higher classification accuracy. Moreover, the SNP data is highly colinear given its genetic proximity in regions of linkage disequilibrium (LD), and therefore the effective amount of SNP is smaller. Therefore, we explored different thresholds to select possible SNPs. In particular, we used the top 100 SNPs and a cut-off of 10^−3^ to select approximately the top 1,000 SNPs for comparison purposes (n = 1,106 SNPs). These SNPs datasets will be referred to as Top100 and T1k, respectively.

### Model building

In summary, the predictive models were built using BSWiMS, GA+SVM+FS, and LASSO. The first performs a robust step-wise model selection using 20 rounds of cross-validations [32]. The second is a genetic algorithm coupled to a support vector machine as a classifier and a final step of forward selection over 1,000 evolved models [33]. For the last [30], we used the implementation in FRESA.CAD, which also uses 20 rounds of cross-validations [32]. All algorithms were tested with the UnrADS subject datasets combined with Top100 and T1k SNPs datasets. Overall, the set used for feature selection consisted of 80% of the dataset (650 AD cases and 732 controls), where the performance was measured in the remaining 20% of samples used as a blind test (163 AD cases and 285 controls).

**Fig 3:**
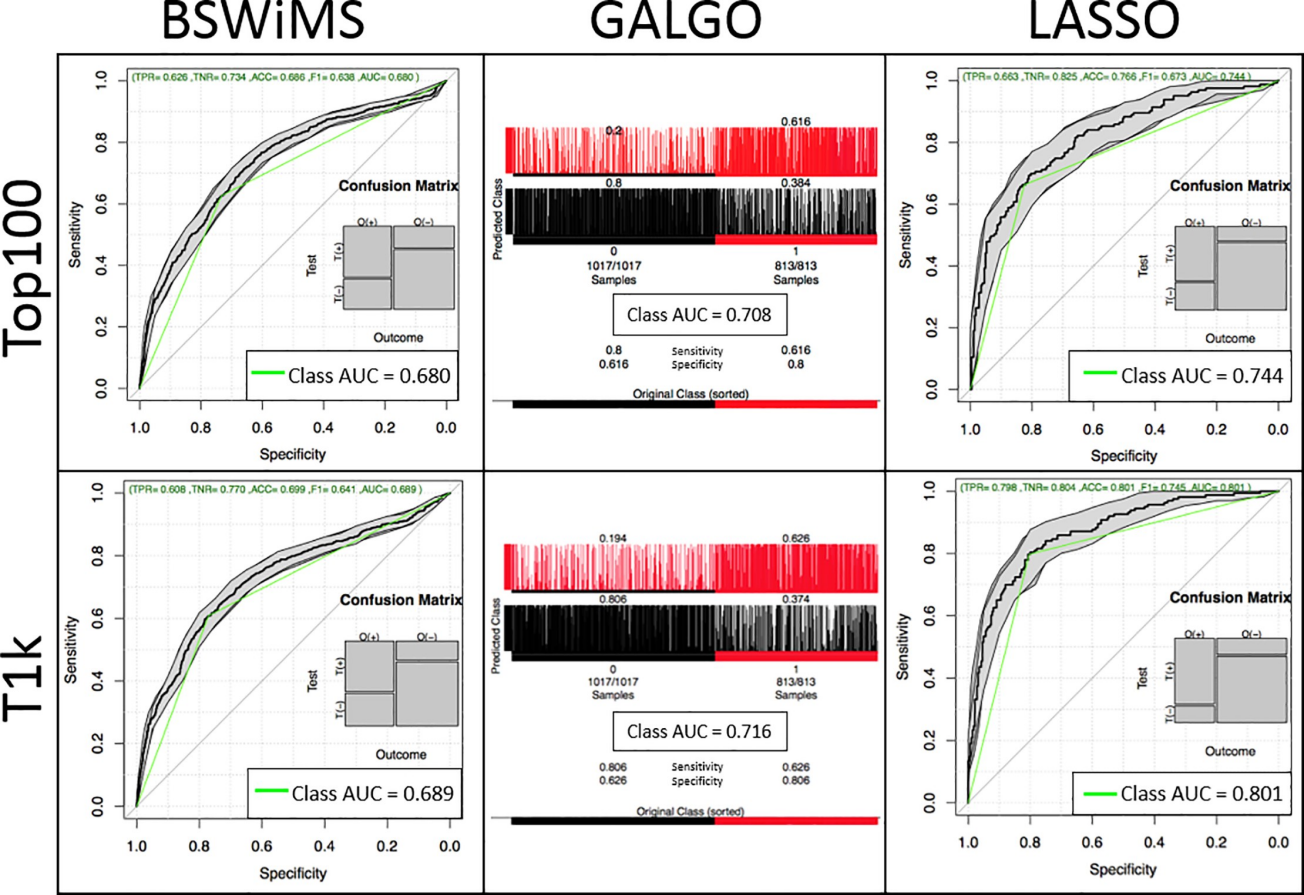
Performance of the three ML methods on two datasets.

Fig 3 displays the performance of the generated models (ROC curves for BSWiMS and LASSO and confusion matrices for GA), while Table 1 summarizes the results. Overall, in terms of accuracy and AUC, the best method was LASSO, followed by GA, then BSWiMS. Nevertheless, in terms of model length, the best method was BSWiMS, followed by GA, then LASSO. Across the three methods, using 1,106 SNPs improved the performance than using only 100 SNPs, suggesting that some SNPs in T1k are informative and were indeed required by all methods to improve the performance. However, the generated model was not always longer; in GA, the yielded model was reduced from 49 to 42 while the AUC increased from 0.708 to 0.716. In LASSO, the model size increased drastically from 71 to 433 SNPs suggesting that many SNPs are included but with a marginal contribution. Nevertheless, the improvement in performance was substantial, from 0.744 to 0.801. For LASSO, we repeated the experiment by selecting a different member of the family randomly, and the results were similar (AUC = 0.820), suggesting than particular family members are indistinct.

**Table 1:** Summary of model building for all methods tested.

Model	SNP Dataset	Input SNPs	Model Length*	Accuracy	Sensitivity	Specificity	AUC
**BSWiMS**	Top100	100	32	0.686	0.626	0.734	0.680
	T1k	1,106	55	0.699	0.609	0.770	0.690
**GALGO**	Top100	100	49	0.720	0.616	0.800	0.708
	T1k	1,106	42	0.726	0.626	0.806	0.716
**LASSO**	Top100	100	71	0.766	0.663	0.825	0.744
	T1k	1,106	433	**0.801**	**0.798**	**0.804**	**0.801**

*The number of features included in the generated model.

**Fig 4:**
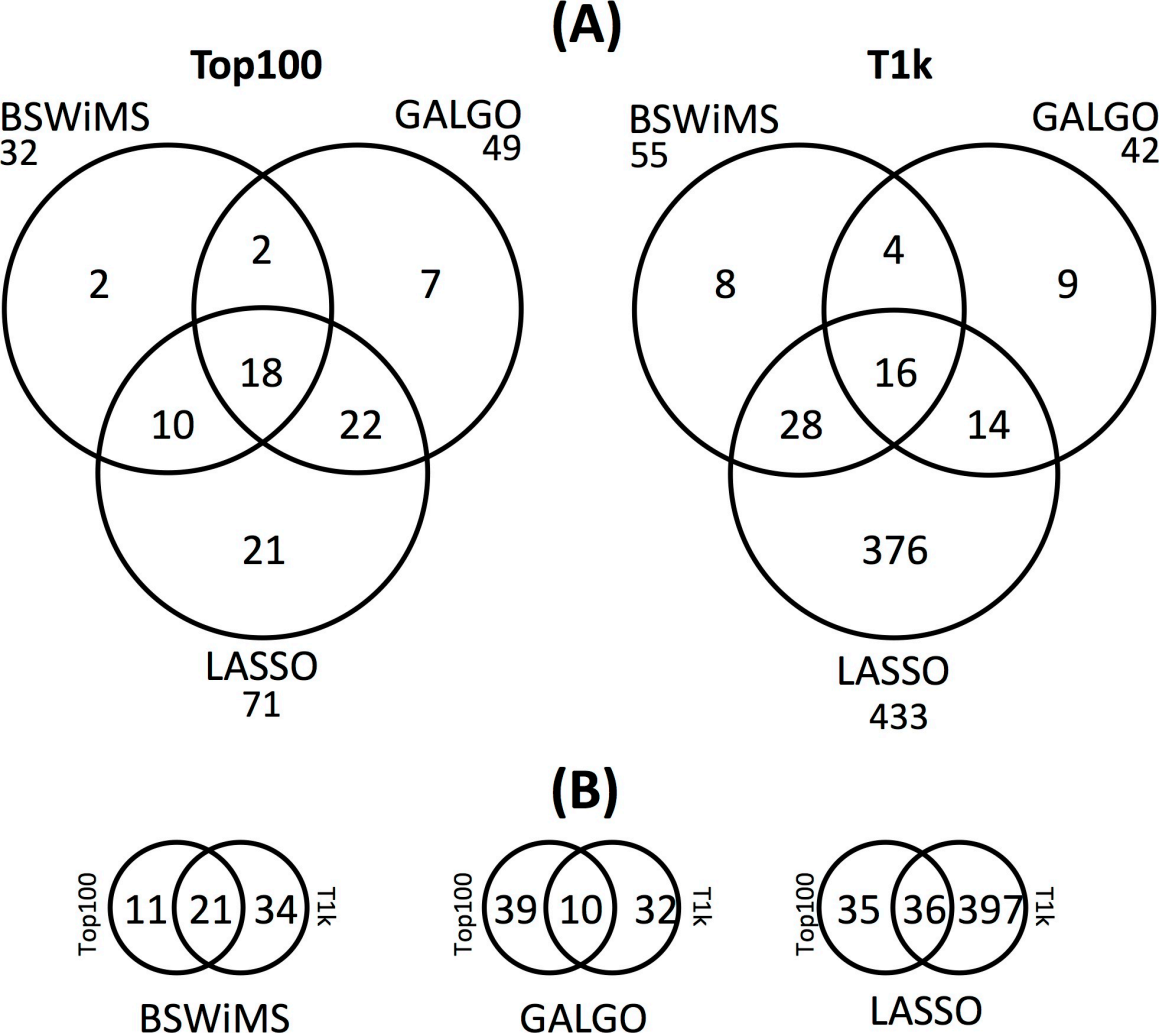
Comparison of the SNPs obtained from the three ML methods. Details are shown in supplementary table.

To analyze the similarity of the models, we compared the number of shared SNPs from the models, as shown in Fig 4A. The details of the included SNPs are shown in supplementary tables (S1 to S6 Tables). In general, most of the SNPs were shared between models suggesting that few polymorphisms are method-specific. Nevertheless, for the LASSO model in the *T1k* dataset, which was the largest generated model, there were 376 method-specific SNPs. This estimation is not largely affected by the 13 SNPs highly correlated (r^2^ > 0.95 from the 433 set) that may provide alternative SNPs. We then compared the SNPs included by the same method across the *Top100* and the *T1k* datasets. The results represented in Fig 4B show that the SNPs included are highly variable across methods suggesting that the inclusion of SNPs is highly conditional on the method and the presence of other SNPs.

### Re-modeling of misclassified samples

**Fig 5:**
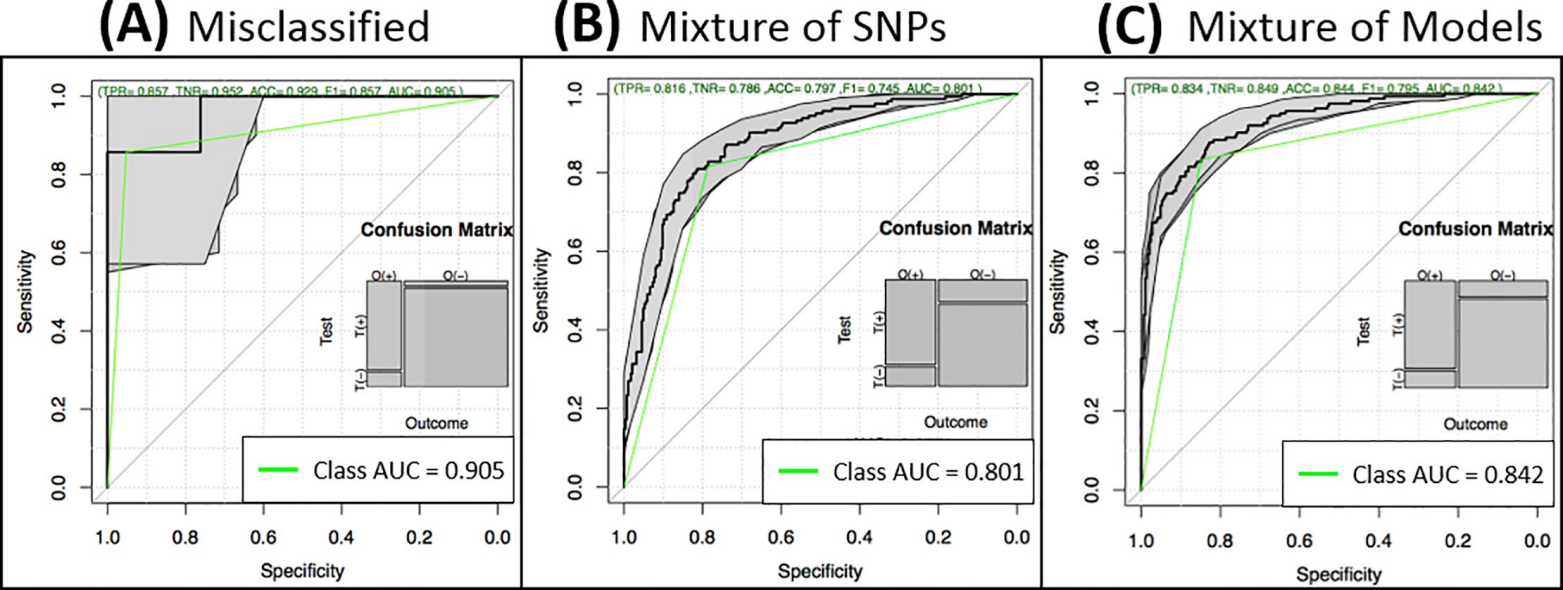
Incorporation of misclassified samples to the predictive model. (A) Performance of the misclassified samples. (B) Performance of the model generated by using the SNPs in T1k and the SNPs from misclassified samples as input for model building. (C) Performance of the model generated by using the SNPs from the T1k LASSO model of Table 1 and the misclassified model as input for model building.

We assumed that misclassified samples do not share the same information as those correctly classified. Thus, we used the 89 misclassified testing samples (56 AD cases and 33 controls) of the LASSO model of Table 1 or the 21 misclassified training samples (9 AD cases and 12 controls) to pre-select SNPs from WADS using the χ^2^ test with kinship correction. Consequently, 461 or 384 SNPs were chosen from test or train sets, respectively. Then we re-built a LASSO model. We focused on LASSO because it was the best method in the above results. The results show surprisingly high performance in these particular small sets reaching an AUC of 0.905 and 0.875 (respectively to test and train) and an accuracy of 0.929 and 0.833 (test and train respectively), as displayed in Fig 5A and Table 2 (rows *missed*). These results, and their consistency in both sets, confirm that there are specific polymorphisms in *missed* subgroups of individuals and may, therefore, be the result of differences with the largest group of samples.

**Table 2:** Model building before (T1k) and after re-modeling of misclassified samples (Mix) using lasso.

SNP Dataset	Samples (AD, Ctrl)	Input SNPs	Model Length	Accuracy	Sensitivity	Specificity	AUC
T1k*	448 (163, 285)	1,106	433	0.801	0.798	0.804	0.801
Missed (test)*	*89 (56*, *33)*	*461*	*49*	*0*.*929*	*0*.*857*	*0*.*952*	*0*.*905*
SNPs Mix*	448 (163, 285)	1,567	493	0.797	0.816	0.786	0.801
Models Mix*	448 (163, 285)	482	358	**0.844**	**0.834**	**0.849**	**0.842**
T1k *(train)*	1,382 (650, 732)	1,106	433	0.985	0.984	0.986	0.985
*Missed (train)*	*21 (9*, *12)*	*384*	*8*	*0*.*833*	*1*.*000*	*0*.*750*	*0*.*875*
SNPs Mix*	448 (163, 285)	1,490	494	0.813	0.816	0.811	0.813
Models Mix*	448 (163, 285)	501	391	**0.848**	**0.828**	**0.860**	**0.844**

* Indicates that the evaluation was performed in the test set. AD, CTRL refer to Alzheimer’s Disease and Control subjects.

### Incorporating model of misclassified samples

We explored two methods to generate an overall model from the *T1k* LASSO model shown in Table 1 and the *Missed (test)* model of misclassified samples shown in Table 2. The first method consisted of mixing the input SNPs from both models and let LASSO and the internal cross-validation of the used package to select the best combination of SNPs. Thus, the input consisted of 1,567 SNPs (1,106 SNPs from the *T1k* model plus 461 SNPs from the *Missed* model) using the same UnrADS samples. Fig 5B and Table 2 (first row *SNPs Mix*) demonstrate that the model generated did not improve in terms of AUC. Besides, the model increased the number of SNPs used from 433 to 493. The second method consisted of mixing the SNPs selected in both models as the input to LASSO and the internal cross-validation. Thus, the input included 482 SNPs (433 from the *T1k* model of Table 1 plus 49 SNPs from the *Missed (test)* model of Table 2) also using the UnrADS samples. Fig 5C and Table 2 (first row *Models Mix*) demonstrate that the AUC increased from 0.801 to 0.842. Moreover, the model decreased the total number of SNPs needed from 433 to 358 (S7 Table). This result suggests that SNPs specific for misclassified samples help to improve predictive models for AD. The same procedure applied to *Missed* samples in the training set also produced an improved AUC (0.844) and a reduced set of SNPs (391). Therefore, gaining information from missed samples, either in the train or test sets, increase the prediction of models.

## Discussion

In this research, we aim to explore other methods to generate polygenic models to predict Alzheimer’s disease. Although it is known that age and gender are important predictors of AD (for example [19]), we are focused on predicting AD from GWAS data only to show a potential computational strategy. In this context, using GWAS only, we improved about 5% on AUC by using specific information from misclassified samples either in the train or test sets. This result has some implications. First, it suggests that misclassified samples are methodologically overlooked, which happens mainly when misclassified samples are a minority of the cases because statistic and computational algorithms attempt to maximize classification focusing on the majority of them. Contrary to this case, for example, are the non-APOE (e4 or e2) risk carriers, where the PRS is still predictive at a similar level [18], which shows that risk alleles included in the PRS are, somehow and not surprisingly, independent. Thus, the misclassified samples seem to be a different genetic sub-class. Because of our data processing and filtering, this sub-class should not be related to marked ethnic differences. Second, because the misclassified samples are a minority, more samples of this sub-class would be needed for more proper analyses. In our analyses, only 89 samples were misclassified from the test or train set and re-used as feedback to obtain a polished model. Indeed, this also influences the train-test scheme. Third, given that incorporation of predictive SNPs from misclassified samples improved the overall predictive power, it suggests that other methodological strategies could be further explored, such as a tree of models where first nodes attempt to identify the right model to use. This may have an influence on designing predictive models for AD. Fourth, it also raises questions regarding the SNPs specific of the misclassified samples. For example, are these SNPs associated with AD? or are methodological artifacts?

A recent meta-analysis used a PRS to assess the prediction of AD based on GWAS, generating an AUC of 0.827 [14]. Here, as a proof of concept, we report an improvement to ~0.84. Nevertheless, some differences need to be highlighted. First, the size of the database used by us is by almost two orders of magnitude smaller, resulting in less heterogeneity, more difficulties in finding informative SNPs, and unfortunately, less generalization. Second, their prediction was reached on a much larger universe of SNPs using those whose p < 0.5, while our models are based on less than 500 SNPs. Third, we gained information from misclassified samples.

A limitation of our study is the use of a very relaxed threshold to select SNPs. This is due to the limited number of highly significant SNPs and the familial nature of the dataset used. Other larger datasets may help to more properly select candidate SNPs, such as those used in meta-analyses [14].

## Conclusions

We presented a direct comparison of step-wise and genetic algorithms and L1 (LASSO) methods to build logistic models for the prediction of Alzheimer’s disease. To the best of our knowledge, this is the first study that compares these methods on GWAS data to generate predictive models for Alzheimer’s disease. LASSO models were more predictive than the other methods in our experiments. Moreover, and unlike other studies, this work successfully incorporates the analysis of poorly classified samples in predictive models to increase prediction. Hence an improved picture of the polymorphisms associated with the risk of AD prediction was produced. We observed that adding information from misclassified AD and control cases, either from train or test, generate more powerful models suggesting future strategies for risk prediction. Our models finally used less than 500 SNPs, and final predictive models comprised fewer SNPs than original models, not considering misclassified samples. The best strategy to generate the predictive models involved the LASSO re-run using the mixture of the SNPs obtained from the original model plus the SNPs obtained from the model of misclassified samples. The AUC obtained improved over recent meta-analysis [14].

## Supporting information

S1 Fig Data quality procedures. (A) Sample processing (B) SNP processing.
(TIF)

S1 Table SNPs included in the BSWiMS model for the Top100 dataset. (CSV)

S2 Table SNPs included in the GALGO model for the Top100 dataset. (CSV)

S3 Table SNPs included in the LASSO model for the Top100 dataset. (CSV)

S4 Table SNPs included in the BSWiMS model for the T1k dataset. (CSV)

S5 Table SNPs included in the GALGO model for the T1k dataset. (CSV)

S6 Table SNPs included in the LASSO model for the T1k dataset. (CSV)

S7 Table SNPs included in the LASSO model from mix-models for the T1k dataset. (CSV)
