# Supplementary figures and images for: Improving predictive models for Alzheimer’s disease using GWAS data by incorporating misclassified samples modeling
Source: PLoS One. 2020 Apr 23;15(4):e0232103. doi: 10.1371/journal.pone.0232103 (PMC7179850; doi:10.1371/journal.pone.0232103)

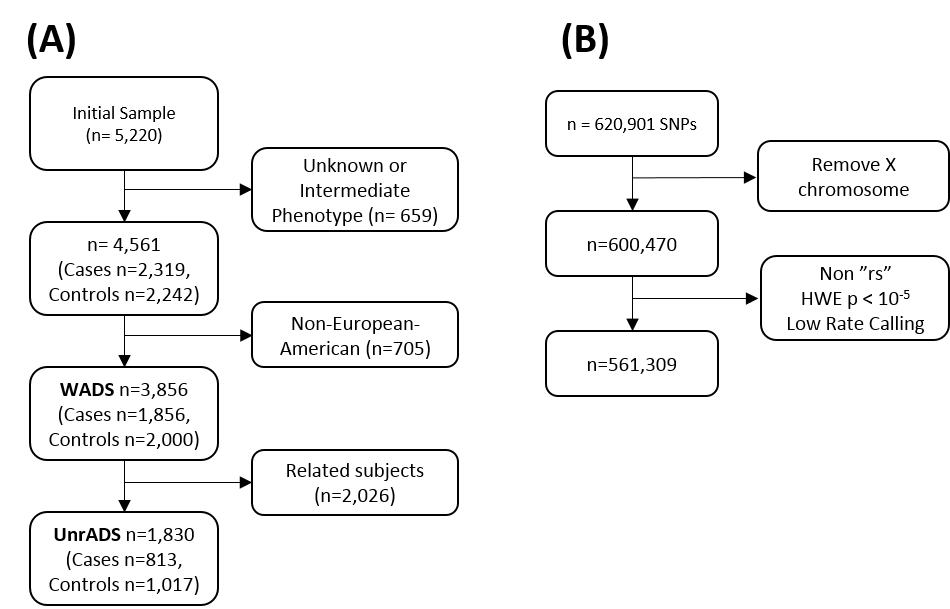

Supplement: S1 Fig — (A) Sample processing (B) SNP processing. (TIF) [file pone.0232103.s001.tif]
